# Anti-Inflammatory Effects of Urocanic Acid Derivatives in Models *Ex Vivo* and *In Vivo* of Inflammatory Bowel Disease

**DOI:** 10.5402/2012/898153

**Published:** 2012-09-05

**Authors:** Arthur Kammeyer, Charlotte P. Peters, Sybren L. Meijer, Anje A. te Velde

**Affiliations:** ^1^Department of Dermatology, Academic Medical Center (AMC), University of Amsterdam, P.O. Box 22700, 1100 DE Amsterdam, The Netherlands; ^2^Tytgat Institute for Liver and Intestinal Research, Academic Medical Center (AMC), University of Amsterdam, P.O. Box 22700, 1100 DE Amsterdam, The Netherlands; ^3^Pathology Department, Academic Medical Center (AMC), University of Amsterdam, P.O. Box 22700, 1100 DE Amsterdam, The Netherlands

## Abstract

Urocanic acid (UCA) derivatives were tested for their anti-inflammatory activity in inflammatory bowel disease (IBD) in two models: *ex vivo* and an experimental mouse model. *Ex vivo*: inflamed colonic tissue was incubated in culture medium with or without the UCA derivatives. Biopsies, incubated with UCA derivatives, produced lower levels of proinflammatory cytokines IL-6 and IL-8 as compared to control biopsies. The same compounds also showed increased levels of IL-10, providing an additional indication for anti-inflammatory properties. *In vivo*: a combination of two imidazoles and a combination of two of their ethyl esters were administered to mice while colitis was induced by oral administration of dextran sodium sulfate (DSS). Some parameters did not show conclusive effects, but the imidazoles and their ethyl esters reduced the area of inflammation and the number of infiltrating neutrophils. Fibrosis and the sum of all histological aspects were reduced by the imidazoles, whereas the ethyl esters reduced the colon weight to length ratio. These results suggest that the UCA derivatives have anti-inflammatory effect on IBD. In addition, fine tuning of the *ex vivo* model may provide an elegant way to predict anti-inflammatory effects of potential drugs in humans, which may decrease the need for animal experiments.

## 1. Introduction

Inflammatory bowel diseases are chronic inflammatory diseases of the gastrointestinal tract resulting from a complex interaction of genetic, immunologic, and environmental factors. Two major forms of IBD are described: Crohn's disease and ulcerative colitis.

Increased numbers of activated immune cells are present in the intestinal mucosa of IBD patients, resulting in enhanced cytokine levels of tumor necrosis factor-*α* and interleukins (IL), such as IL-6, IL-17, IL-10 [1], and IL-8 [2]. These cytokines are important mediators for interaction between immune, epithelial, and mesenchymal cells [3].

Biologic agents, antagonizing TNF-*α*, are effective in IBD patients to induce and maintain disease remission. However, not all patients benefit from this strategy [4, 5]. Other current approaches, such as biologic agents antagonizing proinflammatory cytokines (e.g., IL-6) or the ones that favour increased levels of the suppressive cytokine IL-10, did not result in a substantial reduction of IBD disease activity in clinical trials [6].

Therefore, new therapeutic strategies are urgently required that may be based on small-molecular compounds, rather than biologic agents. Here we present a new class of small-molecular compounds, the UCA derivatives that originated from UV-exposure of the skin. The incidence of inflamed bowel diseases (IBD) varies according to geographical location, that is, near the equator there is a smaller incidence than in northern countries in Europe and North America [7–11]. Exposure to solar UV near the equator is more intense and is known to cause immunosuppression or anti-inflammatory effects [12]. An inverse correlation may exist between daily UV-doses and the severity of IBD.

One proposed mechanism is related to the occurrence of relatively high concentrations of *trans*-UCA in the stratum corneum. The absorption of UV-radiation by this compound leads to a partial isomerisation to *cis*-UCA, or to (photo) oxidation yielding UCA oxidation products [13]. Both have shown to have anti-inflammatory effects both *in vivo* and *in vitro* [14–16]. In the case of *cis*-UCA, attenuation of experimental colonic epithelial damage, induced by dextran sodium sulfate (DSS), has recently been obtained *in vivo* [17].

Urocanic acid (UCA) derivatives, studied here, are natural and synthetic derivatives of UCA. Several UCA derivatives are potential anti-inflammatory agents, primarily developed for topical applications on inflamed skin [15, 16].

Two natural UCA derivatives, imidazole-4-carboxylic acid (ImCOOH) and imidazole-4-acetic acid (ImAc), were found to have an excellent safety profile after the preclinical test phase, including partial systemic exposure through skin penetration (unpublished report). The natural derivatives of UCA are photo (oxidation) products of UCA and can occur in low concentrations in UVB-exposed skin, for example, skin exposed to bright sun [13]. Synthetic UCA derivatives are derived from their natural precursors and were alkylated to obtain enhanced lipophilicity. A smoother passage through biological membranes and increased penetration through the skin are expected. Their safety profiles are not yet defined. Two ethyl esters of the synthetic derivatives were included.

Together, six UCA derivatives were tested for anti-inflammatory effects on inflamed human colon tissue, derived from patients with ulcerative colitis in an *ex vivo* model of ulcerative colitis. Two amide-like UCA-derivatives were included in the *ex vivo* experiments next to two ethyl esters and the natural compounds ImCOOH and ImAc. An anti-TNF-antibody was included in the *ex vivo* experiments as well, to compare effects of the UCA derivatives with a well-established anti-inflammatory agent.

In addition, an experimental DSS mouse model was used to study the anti-inflammatory effects of the UCA derivatives on the reduction of DSS-induced epithelial damage and the relation between both experimental approaches is evaluated.

## 2. Materials and Methods

### 2.1. Test Compounds

Test compounds imidazole-4-carboxylic acid (ImCOOH) and imidazole-4-acetic acid (ImAc) were synthesized by Chemshop BV. Ethyl imidazole-4-carboxylate (ImCOOEt) was supplied by Combi Blocks Inc. and imidazole-4-carboxamide (Im-carboxamide) was obtained from Santa Cruz Biotechnology. Imidazole-4-acetamide (Im-acetamide) and ethyl imidazole-4-acetate (Et-ImAc) were synthesized in our laboratory, starting with esterification of imidazole-4-acetic acid and ammonialysis to obtain the acetamide. The anti-TNF antibody, known as infliximab, was obtained from Schering-Plough/MSD. Dextran sodium sulfate (DSS) was supplied by TdB Consultancy. The immunohistological staining of neutrophils was performed on snap frozen sections with a primary rat anti-mouse Ly-6B.2 antibody (clone 7/4, number MCA771GT, AbD Serotec, followed by a secondary antibody donkey anti-rat horse radish peroxidase (Jackson Immunoresearch Laboratories)). Color development proceeded with a staining kit, based on 3,3′-diaminobenzidine tetrahydrochloride (Dako North America Inc.).

### 2.2. Test Compounds Solutions for *Ex Vivo* Experiments

**Figure 1:**
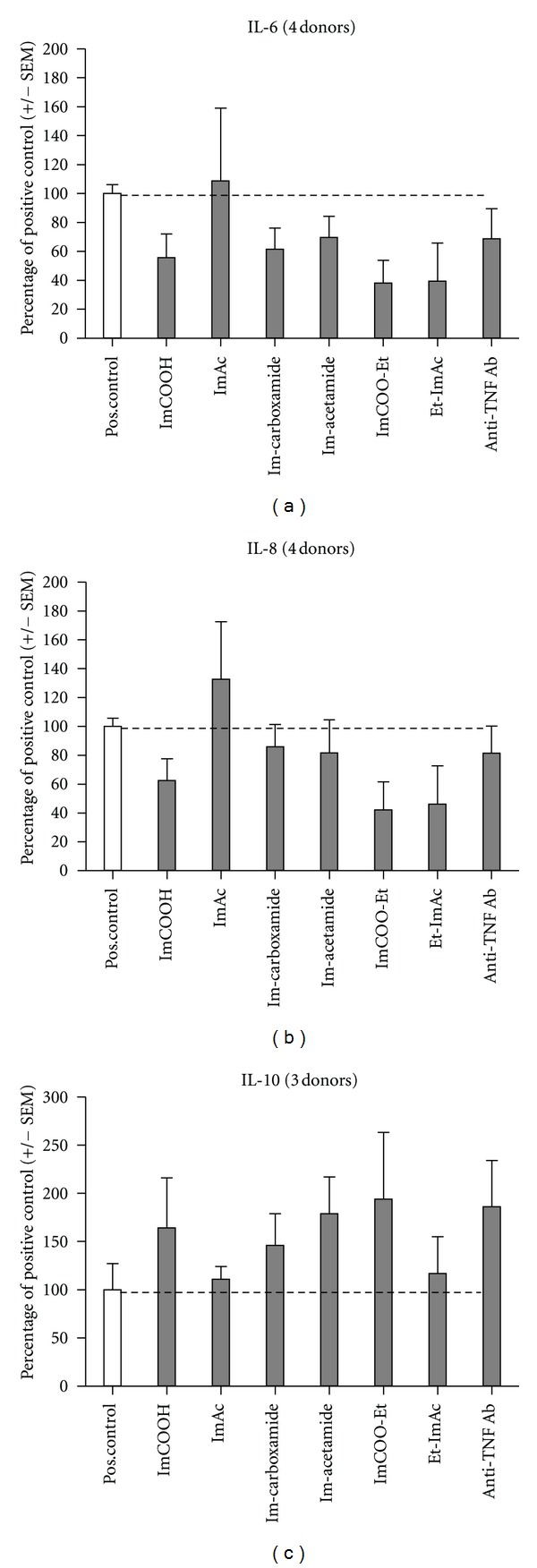
Cytokine levels of IL-6, IL-8, and IL-10 found in the experiment *ex vivo* with ulcerative colitis tissue, derived from coloscopy. The cytokine levels found with only the addition of PBS were set to 100%. The cytokine levels, obtained from test compound additions, were related proportionally.

The UCA derivatives ImCOOH, ImAc, Im-carboxamide, Im-acetamide, ethyl imidazole-4-carboxylate (ImCOOEt), and ethyl imidazole-4-acetate (Et-ImAc) were dissolved in a concentration of 100 mmol/L in phosphate-buffered saline (PBS). In the case of ImCOOH and ImAc, the pH value was readjusted to 7 with a sodium hydroxide (NaOH) solution. All solutions were argon saturated and kept under an argon atmosphere until use, because the stability of UCA derivatives towards oxygen has not been fully defined. Each UCA derivative was added at a final concentration of 2 mmol/L. Argon-saturated PBS was added to the medium to obtain a positive control sample, reflecting nonmodified cytokine patterns, and set to 100% in Figure 1.

### 2.3. Test Compound Solutions for the DSS Experiment

UCA derivatives were dissolved in argon-saturated, oxygen-poor PBS. Intraperitoneal (i.p.) injections of 0.2 mL of combined ImCOOH (85 mM) and ImAc (85 mM), referred to as “imidazoles”, each at a dose of 0.85 mmol/kg, were given to one group and 0.2 mL of combined ImCOOEt (42.5 mM) and Et-ImAc (42.5 mM), referred to as “ethyl esters”, each at a dose of 0.425 mmol/kg, to a separate group. As a positive control, 0.2 mL PBS i.p. was administered to a third test group of mice.

Stock amounts of UCA derivative solutions were freshly prepared on day 1 and day 4 of the mouse experiment. Stability checks on UCA derivative solutions were performed with HPLC, in particular for monitoring eventual hydrolysis of the ethyl esters. The analyses showed sufficient stability across the period of use (3-4 days), with minor hydrolysis of Et-ImAc into ImAc (approx. 4%). ImCOOEt did not show detectable hydrolysis.

### 2.4. Animals

Female C57Bl/6 mice of 7 weeks were purchased from Charles River (L'Arbresle, France). Three weeks prior to the start of the experiment, three groups of 10 mice were divided across separate boxes, kept in light-, humidity-, and temperature-controlled rooms in the in-house animal facility. They were given water, containing 1.5% DSS, *ad libitum, *and CRM-E food of Special Diets Services (SDS, Witham, Essex, UK). Approval of the Animal Welfare Committee was obtained for all DSS experiments performed (approbation number DIX102231).

### 2.5. Cytokine Quantification

Cytokines IL-6, IL-8, and IL-10 were quantified in the medium obtained from the *ex vivo* culture experiments by means of ELISA (Quantikine, R&D systems).

### 2.6. *Ex Vivo* Model of Inflamed Colon Tissue

Inflamed colon tissue from IBD patients was obtained as residual material from clinical procedures according to the ethical guidelines of the Academic Medical Center (AMC), Amsterdam, The Netherlands. Tissue was subdivided into small pieces of 10–20 milligrams with the aid of a skin biopsy puncher (4 mm ø), followed by submersion in 200 *μ*L RPMI culture medium, containing fetal calf serum (10%), L-glutamine (1%), and penicillin (1 U/mL)/streptomycin (1 *μ*g/mL).

Test compound solution (100 mmol/L) was added to 200 *μ*L medium to obtain a final concentration of 2 mmol/L, followed by a 24-hour incubation period at 37°C in an atmosphere containing 5% CO_2_. Subsequently, the supernatant was collected (approx. 180 *μ*L), centrifuged to remove debris, and frozen at −20°C until further use for cytokine quantification.

### 2.7. Experimental Procedure of DSS Experiment

Mice received 1.5% DSS in drinking water on days 0–7. The weight of each drinking bottle, as well as body weight of the C57Bl/6 mice, was noted each day. Intraperitoneal (i.p.) injections were given once a day on days 1 to 3, followed by twice daily injections on days 4 to 6. Mice were sacrificed on day 7.

### 2.8. Parameters of the DSS Experiment

The colon was removed and freed from surrounding fatty tissue and mesenteric lymph nodes. Entire colon length and weight were measured. Subsequently, the colon was longitudinally dissected into two halves. One half was rolled up on a plastic waver for paraffin embedding and the other half was snap frozen and stored at −80°C.

Disease activity score (DAI) was based on the cumulative scores of three different parameters: stool consistency (0–4), presence of blood in stool (0–4), and categorized weight loss (0–4). The sum was divided by 3.

Histological examination was performed on paraffin-embedded, rolled-up colons, stained with hematoxylin and eosin (HE) and on snap-frozen tissue, immunohistologically stained for Ly-6B.2. A pathologist, blinded for this study, performed the scores for aspects as inflammation involved areas, edema, fibrosis, erosion/ulceration, crypt loss, granulocytes, and mononuclear cell infiltration. The absence of inflammation aspects was assigned as “0”, and the most severe inflammation aspects were assigned as “3”. Scores were summed per slide and per test group of 10 mice, providing an overall score and a score per histological aspect.

### 2.9. Statistics

Statistical evaluation on all parameters was performed using a Kruskal-Wallis test, eventually followed by Dunn's posttest. In one case a Mann-Whitney *t*-test was additionally performed.

## 3. Results

### 3.1. * Ex Vivo* Experiments

Inflamed tissue from patients with ulcerative colitis was used in *ex vivo* experiments. The focus of the experiment was on the following cytokines (i.e., IL-6, IL-8, and IL-10) which are representative of an innate response which is known to be elevated during DSS-induced epithelial damage [1, 3, 6, 12, 14, 17].

The UCA derivative-induced effects on IL-6, IL-8, and IL-10 levels are presented in Figure 1. All UCA derivatives, except ImAc, showed suppressive tendencies on IL-6 and IL-8 levels. An opposite, agonistic effect of ImAc might be apparent on IL-6 and IL-8 levels. The anti-TNF antibody, Im-carboxamide, and Im-acetamide did not seem to affect IL-6 and IL-8 levels much under this test condition.

Interestingly, the anti-inflammatory effect of the UCA derivatives seemed to be confirmed by the effect on IL-10 levels. Here, ImCOOH, both amides, ImCOOEt, and the anti-TNF antibody increased IL-10 levels; however, significance is not reached (*P* > 0.05, Kruskal-Wallis). The *ex vivo* experiments were found to be susceptible for large variations in equally treated samples. To enhance reliability, inflamed ulcerative colitis tissue of 3 to 4 donors was processed in this explorative study.

### 3.2. DSS Experiment

#### 3.2.1. Body Weight

**Figure 2:**
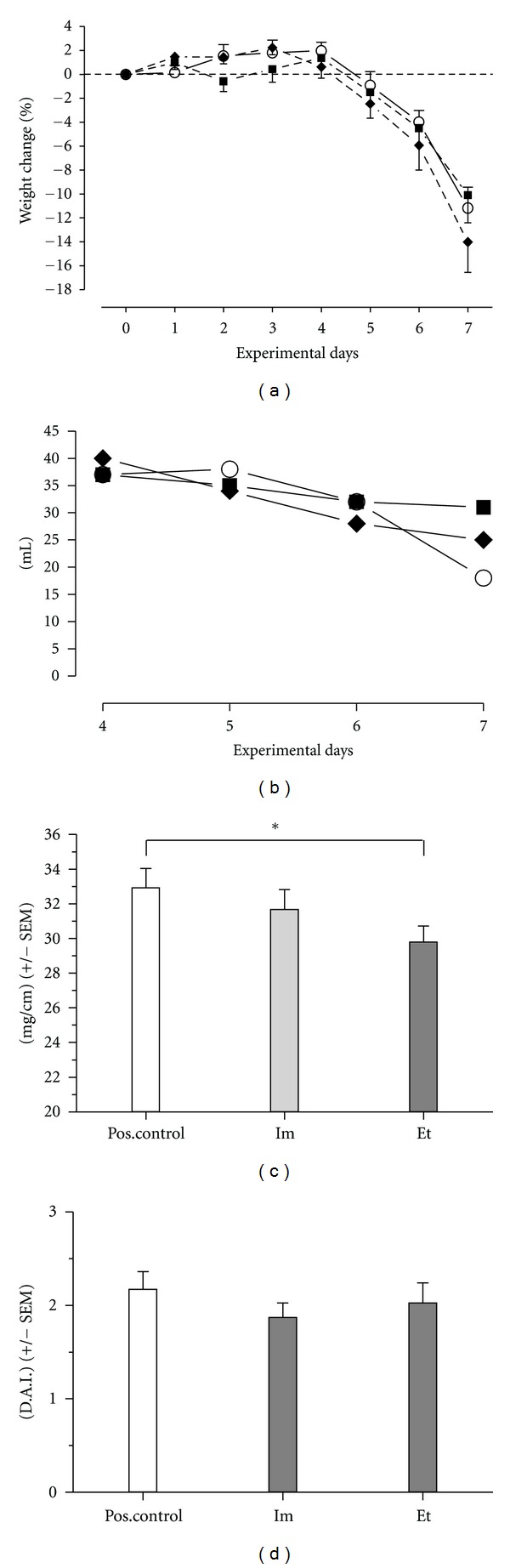
(a) Body weight change during the DSS experiment. Positive control (open circles), treated with the imidazoles (each 0.85 mmol/kg, squares), and treated with the ethyl esters (each 0.425 mmol/kg, canted squares). (b) The monitoring of drinking water consumption during days 4 to 7 of the DSS experiment. Positive control (open circles), treated with the imidazoles (each 0.85 mmol/kg, squares), and treated with the ethyl esters (each 0.425 mmol/kg, canted squares). (c) DSS treatment raises the colonic weight/length ratio. Anti-inflammatory effects will lower the ratio. Colon weight was taken in milligram (mg) and colon length in cm. Pos. control: positive control, the group of mice that only administered PBS intraperitoneally, Im: imidazoles, imidazole-4-carboxylic acid, and imidazole-4-acetic acid in PBS by i.p. injection, Et: ethyl esters, ethyl imidazole-4-carboxylate, and ethyl imidazole-4-acetate in PBS by i.p. injection. *P = 0.036 (Mann-Whitney), however, *P* > 0.05 (Kruskal-Wallis). (d) Disease activity index (DAI), built up from the scores of stool consistency, blood in stool, and categorized weight loss (see Section 2 and (c) for abbreviations).

Body weight of the female C57Bl/6 mice was measured every day at the same time in the morning. The initial weight was set to 0% weight change. Negative values are associated with weight loss (Figure 2(a)). The administration of the UCA derivatives did not show conclusive effects.

#### 3.2.2. Consumption of Drinking Water

The consumption of drinking water was monitored each day of the experiment, and a steep reduction in drinking water consumption was observed in the positive control group during days 6-7, as compared to the two groups receiving the UCA derivatives, who maintained their consumption of drinking water (Figure 2(b)).

#### 3.2.3. Colonic Weight Per Length

The ratio of colonic weight (mg) per length (cm) was calculated as a parameter for disease severity. Decreased ratios indicate anti-inflammatory effects, as can be seen with the administration of the UCA derivatives (Figure 2(c)). The mean weight per cm colon length was reduced in the group of mice receiving both ethyl esters, as compared to the positive control group. Considering a limited number of samples in this explorative study, a Mann-Whitney *t*-test did reveal a significant difference (*P* < 0.05), between positive control and ethyl esters, in contrast to a Kruskal-Wallis multiple comparison test (*P* > 0.05).

#### 3.2.4. Disease Activity Index

The disease activity index (DAI) was determined as outlined in Section 3. A tendency for a lower DAI, induced by the imidazoles (ImCOOH/ImAc), is shown in Figure 2(d), but conclusive effects were not observed.

#### 3.2.5. Blinded Histological Examination

**Figure 3:**
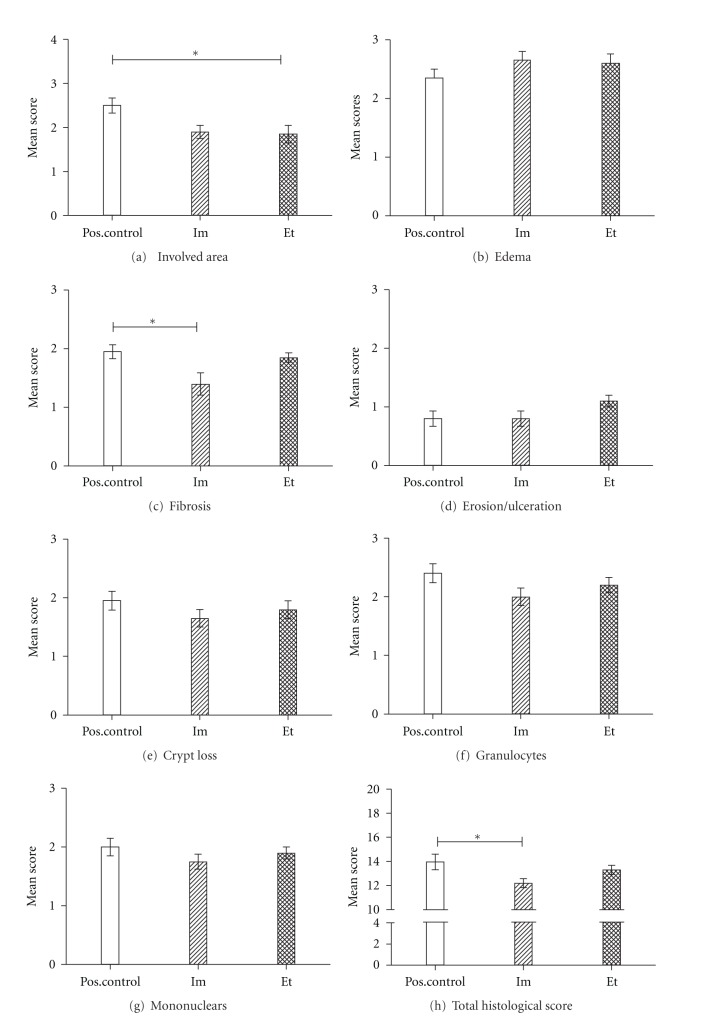
Histological scores (see Section 2) with different parameters. (a) The inflammation involved areas, *P* = 0.01 (Kruskal-Wallis), *P < 0.05 (Dunn's posttest). (b) Severity of edema formation. (c) Occurrence of fibrosis, *P* = 0.02 (Kruskal-Wallis), *P < 0.05 (Dunn's posttest). (d) Occurrence of erosion and ulcerations. (e) Loss of crypt structures. (f) Number of granulocytes, semi-quantitative. (g) Number of mononuclear cells, semiquantitative. The scores of the positive control were compared with those obtained by administration of the test compounds. See for abbreviations Figure 2(c). (h) The summed histological scores of all parameters, *P* = 0.05 (Kruskal-Wallis), *P < 0.05 (Dunn's posttest).

Blinded histological examination of HE-stained paraffin slides were performed according to the histological aspects explained in Section 2. Both individual and total histological scores were documented. The administration of the ethyl ester combination showed a significant reduction in the areas of inflammation (Figure 3(a)). However, when observing the other aspects, only the administration of imidazoles (Im) reduced fibrosis in a significant manner (Figure 3(c)). The other parameters tend to be reduced, predominantly in case of the imidazoles (Figures 3(e), 3(f), and 3(g)), together resulting in a significant reduction of the total histological score for the administration of the imidazoles (Figure 3(h)). The degree of edema (Figure 3(b)) and erosion/ulceration (Figure 3(d)) were not affected by the test compounds.

#### 3.2.6. Immunohistological Staining of Neutrophils

**Figure 4:**
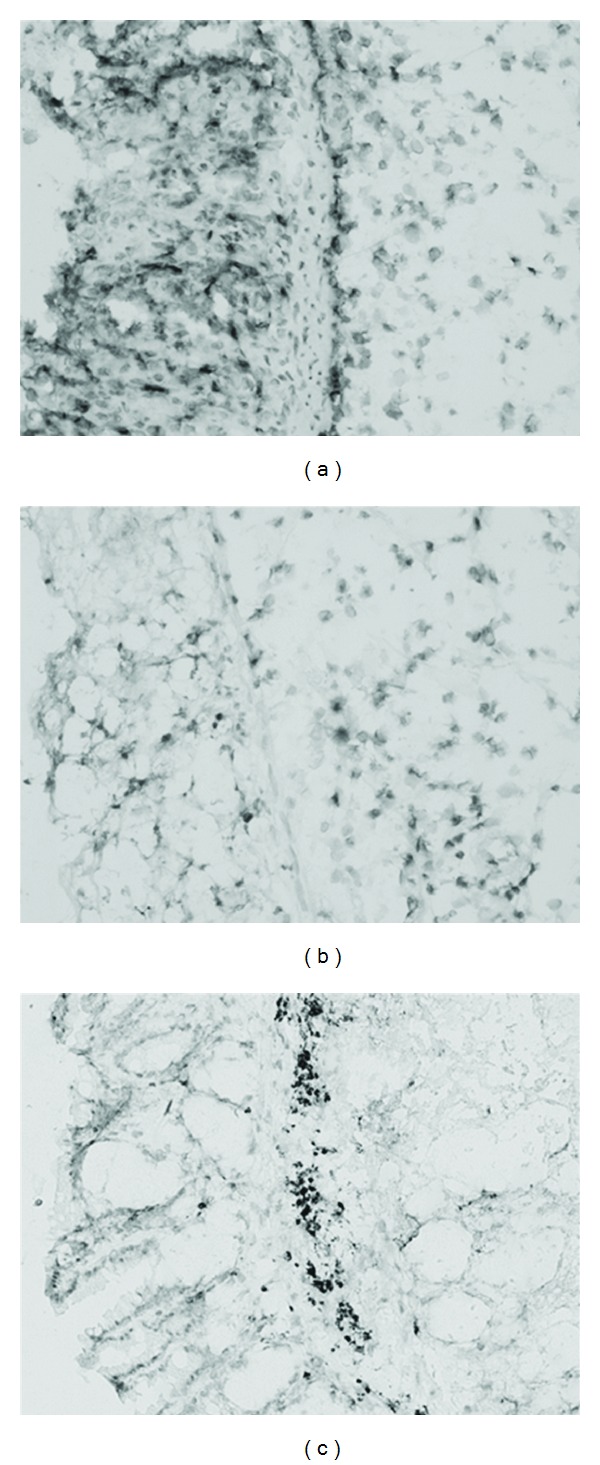
Immunohistological staining of Ly-6B.2 in colonic tissue. From mice receiving: (a) PBS, (b) imidazoles (ImCOOH/ImAc), and (c) ethyl esters (ImCOOEt/Et-ImAc). Magnification 200x. Orientation: epithelial sides are shown on the left. Degrees of inflammation are given in the text of Section 3.

A separate investigation was performed on neutrophil infiltration with immunohistological staining of the Ly-6B.2 epitope on neutrophils. Typical extensive infiltrations are shown in colonic tissue slides of the positive control mice (Figure 4(a)) and a substantially reduced number of neutrophils in colonic tissue of the mice treated with both test compound combinations (Figures 4(b) and 4(c)). Semiquantitative analysis revealed the following degrees of inflammation: ++ for the positive control group (Figure 4(a)), + for the group that administered the imidazoles (Figure 4(b)), and + for the group that administered the ethyl esters (Figure 4(c)) on a scale ranging from +++ (most severe inflammation) to − (no inflammation).

## 4. Discussion

The decrease of inflammatory cytokines (i.e., IL-6 and IL-8) in combination with the increase in IL-10 by the UCA derivatives in the *ex vivo* experiments is encouraging for further development of the UCA derivatives as potential, low-risk anti-inflammatory drug candidate compounds.

The *in vivo* results confirm findings in a recent DSS study that also revealed anti-inflammatory effects on IBD by a UV-induced compound of the skin [17]. Their target compound is *cis*-UCA, the UV-induced stereoisomer of *trans*-UCA [14], and is closely related to the UCA derivatives of this study. Compared to this study, the way of administration and the dosage of *cis*-UCA was different, as well as the mouse strain. In contrast to our study, Albert et al. obtained anti-inflammatory effects with daily subcutaneous injections of 0.36 *μ*mol (50 *μ*g) *cis*-UCA, whereas we injected intraperitoneally 17 *μ*mol of each imidazole and 8.5 *μ*mol of ethyl ester.

Several anti-inflammatory effects were found, indicating that effects of UV-exposure of the skin may attenuate IBD. Remarkably, Albert et al. found a decrease in neutrophil chemoattractant CXCL1, a finding that is in accordance to the reduction of infiltrating neutrophils, shown in this study.

The ethyl esters ImCOOEt and Et-ImAc are expected to have enhanced membrane passage, which property may have enhanced the anti-inflammatory trends, seen in the *ex vivo* experiments. The anti-inflammatory effects of the ethyl esters in the DSS experiment were however not superior possibly due to yet unknown pharmacological events. Routes of administration and delivery of the UCA derivatives at body compartments in general and especially delivery at the mucosal side of the colon should be studied. In that particular layer, permeability and subsequent invasion of antigens are expected to be increased due to the effect of orally ingested DSS.

Oral applications could have been considered to obtain more pronounced anti-inflammatory effects. This was however not possible in the DSS model at this stage. We chose for an intraperitoneal administration route to exclude an unknown intervention with DSS by the oral route. Another model should be used to study the effects of oral application.

The anti-inflammatory agent, infliximab, was introduced in the *ex vivo* tests as a reference. However, the overall effects of the anti-TNF agent are less convincing for anti-inflammatory behaviour under our *ex vivo* test conditions. Longer incubation times may have shown larger anti-inflammatory effects. The documented use for this agent in IBD patients indicates that a longer interaction time is needed for the anti-inflammatory effects to appear [18].

ImAc showed agonistic effects in the *ex vivo* experiments and may have counteracted on ImCOOH, while in the DSS experiments during their combined administration. This late conclusion can be drawn from the *ex vivo* results, where ImAc seems to increase IL-8 levels (Figure 1). Consequently, the anti-inflammatory effect of ImCOOH could have been dampened by ImAc. If indeed true, the *ex vivo* results correlate with the results obtained *in vivo*.

The *ex vivo* model could add value to the question of the usefulness of anti-inflammatory candidate compounds for drug development. If the variation in the results could be decreased, an improved *ex vivo* model may reduce the use of experimental animals in the future.

In this study, six different UCA derivatives were tested *ex vivo* and four of them *in vivo*. Many other UCA derivatives with potential anti-inflammatory properties can be synthesized within the context of a submitted patent (International Patent number PCT/NL2008/050367). This family of UCA derivatives may form a novel class of anti-inflammatory agents for IBD.
